# In Situ Neutral System Synthesis, Spectroscopic, and Biological Interpretations of Magnesium(II), Calcium(II), Chromium(III), Zinc(II), Copper(II) and Selenium(IV) Sitagliptin Complexes

**DOI:** 10.3390/ijerph18158030

**Published:** 2021-07-29

**Authors:** Samy M. El-Megharbel, Moamen S. Refat, Fawziah A. Al-Salmi, Reham Z. Hamza

**Affiliations:** 1 Department of Chemistry, College of Science, Zagazig University, Zagazig 44519, Egypt; 2 Department of Chemistry, College of Science, Taif University, P.O. Box 11099, Taif 21944, Saudi Arabia; moamen@tu.edu.sa; 3 Department of Biology, College of Science, Taif University, P.O. Box 11099, Taif 21944, Saudi Arabia; f.alsalmi@tu.edu.sa

**Keywords:** sitagliptin, complexes, spectroscopic, morphology, STZ, diabetes

## Abstract

Magnesium(II), calcium(II), chromium(III), zinc(II), copper(II), and selenium(IV) sitagliptin (STG) complexes—with the general formulas [Mg(STG)_2_(Cl)_2_]·6H_2_O, [Ca(STG)_2_(Cl)_2_], [Cr(STG)_2_(Cl)_2_]Cl.6H_2_O, [Zn(STG)_2_(Cl)_2_], [Cu(STG)_2_(Cl)_2_]·2H_2_O, and [Se(STG)_2_(Cl)_2_]Cl_2_, respectively—were designed and synthesized by the chemical reactions between metal(II, III, and IV) chloride salts with an STG ligand in situ methanol solvent in a 1:2 stoichiometric ratio (metal:ligand). Tentative structures of the complexes were proposed based on elemental analysis, molar conductance, magnetic moments, thermogravimetric analysis, and spectral (infrared, electronic, and ^1^H NMR) data. The particle size and morphological investigation were checked on the bases of scanning electron microscopy, transmission electron microscopy, and X-ray powder diffraction analyses. All the Mg^2+^, Ca^2+^, Cr^3+^, Zn^2+^, Cu^2+^, and Se^4+^ complexes were found to be six-coordinated, wherein the STG ligands act as bidentate chelating agents. This study demonstrates that pancreatic tissues are affected by the induction of experimental diabetes mellitus and clarifies the potential of the synthesized STG complexes, which was found to more significantly improve insulin secretion and the pancreatic and glycometabolic complications of diabetic rats than STG alone.

## 1. Introduction

Metal ions play important roles in our life processes. It is well known that that both cellular and semi-cellular functions involve metals. The vital role of inorganic salts in living systems can be revealed by studying biological and biochemical systems. The chemistry of inorganic compounds is not the “dead chemistry” that lot of people may imagine [1,2,3]. Presently, metal ions are as important components of life as organic compounds. For example, in cells, Mg^+2^ and Ca^+2^ ions play regulatory important roles. In living systems, metallothionine proteins are rich in mineral ions. Cytotoxicity and antagonizing Cd-induced carcinogenesis can be inhibited in vivo using divalent cations of zinc, calcium, and magnesium. The formation of protein complexes involves the transportation of Fe and other metals by blood plasma [1]. Cu is mainly known as a metallic element that is primarily associated with Cu-dependent cellular enzymes. For many diseases, minerals are also used as inorganic medicines. However, the focus in this article is on of metal molecule interactions, as well as the chemistry and structure of metal complexes [1,2,3,4,5,6,7,8,9,10].

Naturally occurring mineral compounds are vital for life [11]. Human bones require calcium. Cellular activities depend on magnesium. Additionally, potassium and magnesium are the most abundant minerals in every cell. Divalent calcium and magnesium, as well as zinc, copper, iron, and manganese, participate in the biological processes of the nucleus, are present in detectable quantities, and are associated with DNA and RNA in the cellular system [12]. The active formation of RNA depends on the concentration of Mg^2+^ or Mn^2+^. Enzymes in both plants and animals depend on energy provided by magnesium. Magnesium is known to provide energy by stimulating the production of adenosine triphosphate (ATP), which provides energy to billions of cells in our bodies. Mg is the catalyst or stimulant for this reaction and countless other physical activities.

Sitagliptin (STG) is the first agent from gliptins that has been used in the USA since 2006 and in Europe since 2009. Gliptins are drugs for the treatment of diabetes mellitus (type II) by inhibiting DPP4, the enzyme that inactivates glucose-dependent insulinotropic polypeptide (GIP). GIP serves as important stimulator of insulin secretion and a regulator of blood glucose concentration. Furthermore, STG is known to possess antioxidant and anti-inflammatory properties [13,14]. Thus, the inhibition of DPP4 leads to decreases of blood glucose levels in diabetic patients [15]. STG, which is a DPP-4i, has been shown to manage apoptosis and oxidative stress in type I diabetics and animal models [16]. The daily treatment of type II diabetes patients with STG has revealed a suitable glycemic control, as well as a significant reduction in glycosylated hemoglobin (HbA1c), with almost no hypoglycemic risk [17]. Researchers evaluated the pancreatic tissues’ safety in the Trials Evaluating Cardiovascular Outcomes with STG (TECOS). They demonstrated a small risk for incidence of pancreatitis with STG therapy. Additionally, the registration of some cases with associated acute pancreatitis pushed the FDA to announce a post-marketing safety warning to STG [18].

Current developments in the design of metal-based therapeutic agents have demonstrated increasingly important research attempts to develop new compounds with fewer toxic side effects [4,5,6,7,8]. The present article aimed to study the chemical structures of STG complexes with Mg^2+^, Ca^2+^, Cr^3+^, Zn^2+^, Cu^2+^, and Se^4+^ using CHN elemental, molar conductance, FT-IR, magnetic susceptibility, electronic UV-Vis, ^1^H NMR, thermogravimetric analysis (TGA/DrTGA), XRD, SEM, and TEM analyses. Additionally, this study was designed to evaluate the effect of STG and STG–metal complexes on oxidative damage induced by STZ in decreasing blood glucose levels.

## 2. Experimental

### 2.1. Chemical Experiments

Commercially available chemicals were purchased from commercial suppliers (Sigma-Aldrich Chemical Company (St. Louis, MO, USA)) and used as received, used without further purification. Additionally, 407.31 g/mol of STG (CAS number: 486460-32-6; C_16_H_15_F_6_N_5_O) with a purity of ≥98% were used.

Each STG ligand (2 mmol) and divalent Mg^2+^, Ca^2+^, Cr^3+^, Zn^2+^, Cu^2+^, and Se^4+^ (1 mmol) were mixed in 50 mL CH_3_OH and refluxed for 2 h. The produced white (Mg^2+^, Ca^2+^, Zn^2+^, and Se^4+^), green (Cr^3+^), and blue Cu^2+^ solutions were evaporated to half their original volume and allowed to precipitate. Filtration, washing with methanol, and drying in a vacuum desiccator over anhydrous CaCl_2_ were carried out for the synthesized complexes.

The C, H, and N percentage determined using Vario EL Fab. The metal content and water percentage were gravimetrically determined. FT-IR spectral data for the synthesized complexes were measured using an infrared Bruker spectrophotometer ranged between 400 and 4000 cm^−1^. Magnetic susceptibility measurements were performed with a SHERWOOD SCIENTIFIC (Cherry Hinton Rd., Cambridge, UK) magnetic susceptibility balance. Conductance was measured with a HACH conductivity meter model in a dimethyl sulfoxide solvent at a concentration of 10^−3^ M for synthesized complexes. ^1^H-NMR was recorded as dimethyl sulfoxide solutions on a Bruker 600 MHz spectrometer using tetramethyl silane as the internal standard. The electronic absorption spectra were recorded in DMSO solvent within a range of 900–200 nm using a UV2 Unicam UV/Vis spectrophotometer fitted with a quartz cell of a 1.0 cm path length. The X-ray diffraction patterns were recorded with an X’Pert PANanalytical PRO, which targeted copper with a secondary monochromate. The surface morphologies for the particles of complexes were visualized using Quanta FEG 250 scanning (SEM) (JEOL, Akishima, Tokyo, Japan) and transmission (TEM) electron microscopes generating a 20 kV accelerating voltage, and the shapes and sizes of these particles were visualized using JEOL JEM-1200 EX II and JEOL 100s microscopy, respectively. The thermogravimetric analysis of the complexes was performed from room temperature to 800 °C using a TGA/DTA–50H Shimadzu thermal analyzer (JEOL, Akishima, Tokyo, Japan).

### 2.2. Biological Experiments

#### 2.2.1. Experimental Animals

Ninety male albino rats (10 rat/each group) weighing about 170–200 g and aged 3 months were kept in clean metal cages while ensuring good ventilation. Male rats were provided healthy diets. All efforts were made to reduce animal suffering during the experimental work. The experimental design was approved by the Taif University Ethical Committee (approval number: 42-0074) and followed international guidelines for animal use and care.

#### 2.2.2. Drugs and Chemicals

A 1 g vial of streptozotocin (STZ) was obtained from Sigma-Aldrich Company. STG was obtained in the form of JANUVIA^®^ tablets (Merck Sharp and Dohme Ltd., Pavia, Italy). Each tablet was ground and then dissolved in 0.5% carboxymethyl cellulose to get a suspension form.

#### 2.2.3. Experimental Induction of Diabetes Mellitus

After the period of adaptation, an STZ solution was freshly prepared and administered to fasted animals via intraperitoneal (i.p) injection directly after preparation (50 mg·kg^−1^) [19], accompanied by a fat-fed diet to induce type II diabetes mellitus according to [20]. The experimental diabetic animals were fed with a high-fat diet for 3 weeks and then i.p. injected with STZ. The diet of experimental animals consisted of 60% fat, 20% carbohydrates, and 20% protein. After the end of this period, the rats fasted for 8 hr and then received a single i.p. injection of STZ (50 mg/kg) that was freshly prepared in citrate buffer (Ph = 4.5). At 72 h after STZ i.p. injection, the fasting blood glucose level of each rat was recorded by using an Accu-Chek glucometer (Roche, Germany). The diabetic rats with blood glucose levels greater that 280 mg/dL were selected as diabetics, and then we made another glucometer estimation after 3 days to ensure hyperglycemia. The treatments were started after diabetes induction and continued daily for 30 consecutive days.

Male albino rats were randomly divided into 9 groups. Group I (control group) was treated with normal physiological saline, group II (STZ group) received a “single dose” of STZ (50 mg·kg^−1^) (i.p.) [1], group III STZ rats were orally treated with STG (10 mg·kg^−1^) [21], group IV (diabetic rats) received a single oral dose of Mg/STG (10 mg·kg^−1^), and groups V, VI, VI, and VII were exposed to STZ (diabetic rats) and then orally treated with Cu/STG, Zn/STG, Ca/STG, Cr/STG, and Se/STG at the previously described same dose.

#### 2.2.4. Blood Samples Collection

With light anesthesia, blood samples from the eye plexus were collected in capillary tubes. The blood samples were centrifuged at 6000 r.p.m for 15 min to perform the biochemical analyses.

#### 2.2.5. Serum Blood Glucose, C-peptide, Insulin, and HbA1c

Blood glucose levels were evaluated with Biodiagnostic commercial kits, and fasting serum insulin levels were evaluated with a rat ELISA kit (ALPCO Diagnostics, Salem, NH, USA). C-peptide (Sigma-Aldrich) and HbA1c (Cusabio Co., Wuhan, China) levels were measured with ELISA kits.

#### 2.2.6. Preparation of Pancreatic Tissue Homogenates

Male rats were ethically decapitated, and their pancreatic tissues were stored for further investigation. A small portion of the pancreatic tissues was used to estimate the antioxidant biomarkers. Pancreatic tissues were homogenized in a cold buffer and centrifuged at 5000 r.p.m for about 2 h. The obtained supernatant was stored at −80 °C.

#### 2.2.7. Determination of Oxidative Stress Markers

The pancreatic tissue homogenates were used to estimate the GRx “reduced glutathione” level, following the work of Sedlak and Lindsay [22]. Malondialdehyde (MDA) content was determined using the method of Ohkawa et al. [23]. The catalase (CAT) level was determined using the method of Beers and Sizer [24].

#### 2.2.8. Histological Analysis of Pancreas Tissues

Pancreatic tissues were fixed in about 10% formalin “neutral-buffered”. Following the fixation, the pancreatic tissue samples were processed and then stained with hematoxylin and eosin for examination under a light microscope.

#### 2.2.9. Statistical Analysis

The collected data were entered into and analyzed by computer using Statistical Package of Social Services, version 27 (SPSS) (IBM, Armonk, NY, USA, 2020). Kruskal–Wallis and Dunn’s multiple comparison tests were used. In all the tests, a *p* value of ˂0.05 was taken as significant.

## 3. Results and Discussion

### 3.1. Chemistry Part

#### 3.1.1. Microanalytical and Molar Conductance Data

**Figure 1:**
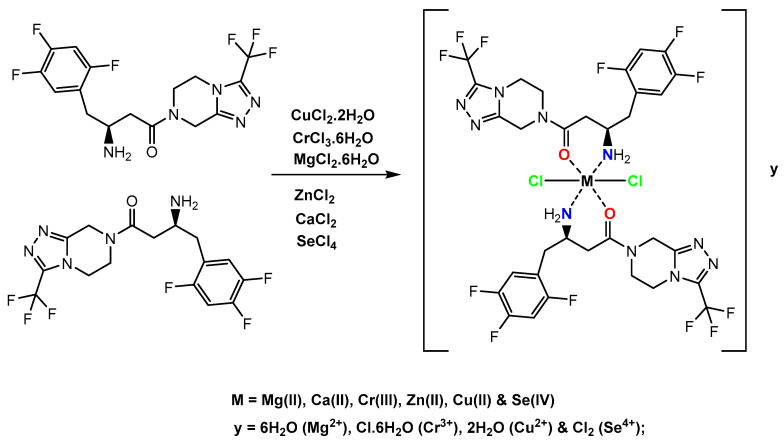
Suggested structures of STG complexes.

These compounds have a high stability at room temperature, low solubility in polar and nonpolar solvents, and a high solubility in DMF and DMSO under gently heating. The conductivity measurements of the magnesium(II), calcium(II), chromium(III), zinc(II), copper(II), and selenium(IV) STG complexes were 25, 32, 76, 29, 33, and 89 μs/cm, respectively. Electrolytic measurements of the trivalent chromium and Se(IV) complexes revealed that their 1:1 electrolytic nature, which suggests that one or two Cl^-^ anions exist outside the coordination sphere [25]. The dissolution of these two synthesized STG complexes gave a white precipitate when mixed with an AgNO_3_ reagent, which confirmed the existence of a Cl^-^ ion in the outer coordination sphere. However, the conductivity measurements of magnesium(II), calcium(II), zinc(II), and copper(II) STG complexes lie in the range of 25–33 μs cm^−1^ at 25 °C. This low conductivity measurement values of the 10^−3^ M concentrated solutions in DMSO showed them to be non-electrolytes. The elemental analysis (Table 1) revealed that the STG complexes have 1:2 metal-to-ligand ratios, as shown in Figure 1.

#### 3.1.2. Electronic Spectra and Magnetic Measurements

The electronic spectra of the copper (II) STG ([Cu(STG)_2_(Cl)_2_].2H_2_O) complex displayed three bands at 12,048, 16,529, and 20,202 cm^−1^ due to ^2^B_1g_→^2^B_2g_, ^2^B_1g_→^2^A_2g_, and ^2^B_1g_→^2^E_1g_ transitions, respectively, which supported the idea that the Cu(II) complex has a distorted octahedral geometry [26,27]. The magnetic moment of this complex was found to be 1.81 BM, which confirmed its octahedral geometry. The assignment of various d–d transitions and charge transfer bands in the spectrum of the [Cr(STG)_2_(Cl)_2_]Cl.6H_2_O complex was performed based on the work of Lever [26] and Lever and Mantovani [27]. For the Cr(III) complex, the electronic spectrum was recorded in DMSO at room temperature. The electronic spectrum of the trivalent chromium complex have three bands at 19231 (ν1), 25974 (ν2), and 41667 cm^−1^ (ν3) corresponding to ^4^A_2g_→^4^T_2g_(F), ^4^A_2g_→^4^T_1g_(F), and ^4^A_2g_→^4^T_1g_(P) transitions, respectively—all of which are characteristics of an octahedral geometry around the Cr(III) ion [28]. The magnetic moment value obtained for the Cr(III) complex was 3.88 BM, which corresponds to an octahedral field. In the case of the STG complexes of Mg(II), Ca(II), Zn(II), and Se(IV), no significant absorption bands in their electronic spectra were obtained due to the diamagnetic nature of these metal ions. The electronic spectra of the divalent magnesium, calcium, zinc, and Se(IV) complexes showed a range of absorption bands at 240–265 nm; these bands can be attributed to the M→L charge transfer transition.

#### 3.1.3. Infrared Spectra

**Figure 2:**
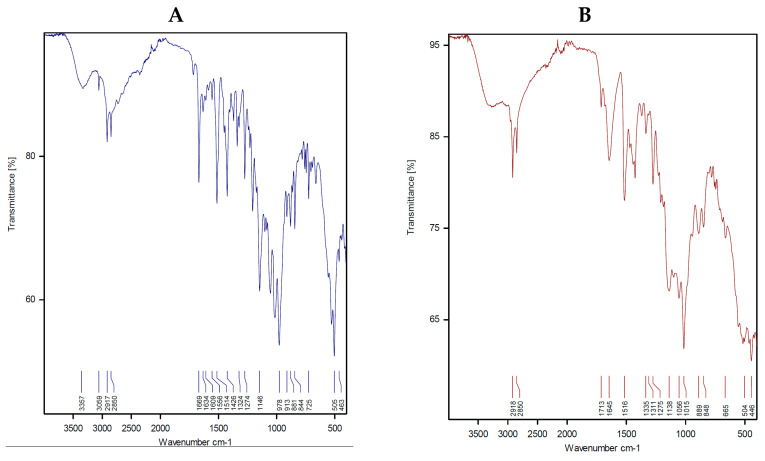

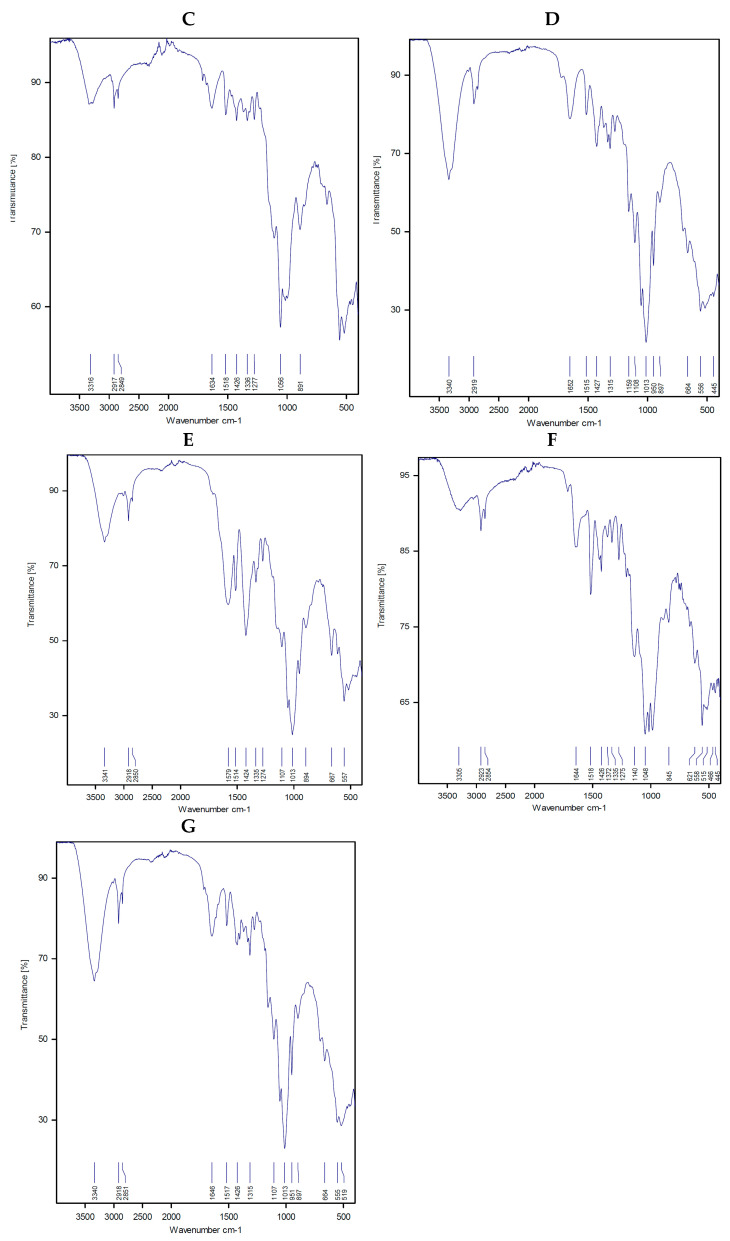
Infrared spectra of the (**A**) STG, (**B**) Mg(II), (**C**) Ca(II), (**D**) Cr(III), (**E**) Zn(II), (**F**) Cu(II), and (**G**) Se(IV) complexes.

The FT-IR spectrum of sitagliptin (Figure 2A and Table 2) showed a number of distinguish bands, such as a strong broad band at 3357 cm^−1^ that can be assigned to the N–H stretching vibration of the NH_2_ group [29,30,31], weak and medium strong bands at 3059, 2917 and 2850 cm^−1^ that can be assigned to the C–H vibration stretching of the aromatic and aliphatic groups, and medium-to-strong bands at 1669 and 1634 cm^−1^ that can be attributed to the υ(C=O) and υ(C=O) vibration stretching of the carbonyl group and 1,2,4-triazole ring, respectively. The strong band at 1609 cm^−1^ can be attributed to N–H bending motion. The three bands at 1556, 1514, and 1426 cm^−1^ can be assigned to the υ(C–N) and υ(C–N) stretching vibrations. The vibrations at 1324 and 1274 cm^−1^ can be attributed to CH in plane bending, whereas the bands at 1146 and 978 cm^−1^ can be attributed to the stretching vibration of the υ(C–F) bond [30,31]. In addition, the characteristic bands at 913, 881, 844, and 725 cm^−1^ can be attributed to the CH out-of-plane bending [29]. The –NH_2_ group stretching vibration was found to be displaced to lower frequency values at 3300, 3316, 3340, 3341, 3305, and 3340 cm^−1^ for the Mg^2+^, Ca^2+^, Cr^3+^, Zn^2+^, Cu^2+^, and Se^4+^ STG complexes, respectively, after chelation. In the FT-IR spectra of the metal complexes, the bands due to the bending vibration of –NH_2_ group disappeared, which indicated the chelation of the STG ligand with the metal ion via an N atom of the NH_2_ group. The vibration stretching of the (C=O) group in the case of the free STG ligand was shown at 1669 cm^−1^ and disappeared in the spectra of STG complexes, which indicated the involvement of the oxygen atom of the C=O group in chelation with metal ions. The band at 1634 cm^−1^ for the STG ligand refers to the stretching vibration of C=N for the 1,2,4-triazole ring, which remained unshifted or slightly shifted to higher wavenumbers in STG complexes, so the nitrogen atom of the C=N group does not participate in the coordination process. The free STG has two stretching bands, at 1146 and 978 cm^−1^ in the C–F region. After complexation, the maxima were found to be the same, suggesting that the C–F bond is far from the place of complexity. The presence of medium-to-weak bands at 558–445 cm^−1^ can be attributed to metal–oxygen and metal–nitrogen stretching vibration motions [29]. Therefore, our infrared spectral results showed that the respective metal ions coordinate with the STG ligand through amino and carbonyl groups and STG acts as a bidentate chelate, as shown in Figure 2B–G.

#### 3.1.4. ^1^H-NMR Spectra

The ^1^H-NMR spectrum of the STG ligand showed two signals in the regions of δ 7.538–7.470 and δ 4.089–4.848 ppm, as well as three signals at δ 2.694–3.981 ppm due to trifluorophenyl ring protons, pyrazine ring protons, and CH_2_ and CH of butan-1-one proton, respectively. The ^1^H-NMR spectrum of the magnesium(II) complex showed signals in the region of δ 8.221–6.670 ppm, which may have been due to four different types of trifluorophenyl ring protons. The signals caused by the pyrazine ring and butan-1-one moiety protons shifted to downfield in the range of δ 4.960–4.259 and δ 4.134–2.803 ppm, respectively, which was due to the decreased electron density and deshielding of protons caused by the participation of the NH_2_ and CO groups in coordination.

#### 3.1.5. X-ray Powder Diffraction Patterns, SEM and TEM Morphological Studies

**Figure 3:**
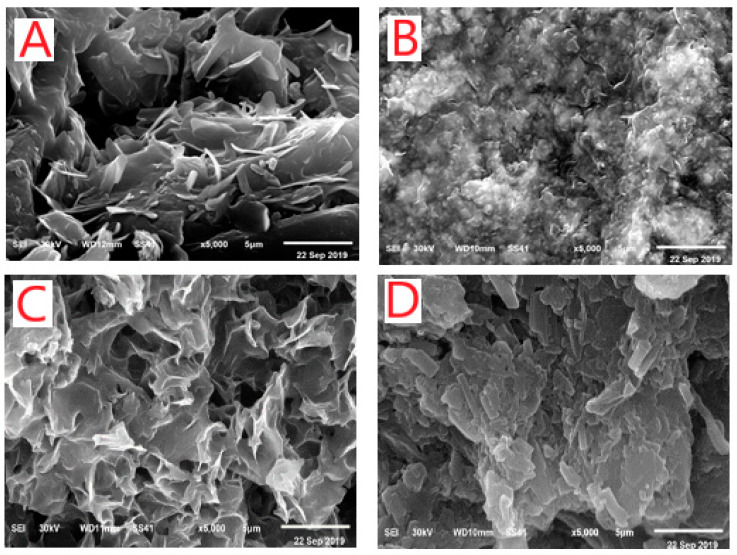
SEM images of (**A**) Mg(II), (**B**) Zn(II), (**C**) Cu(II), and (**D**) Se(IV) STG complexes.

**Figure 4:**
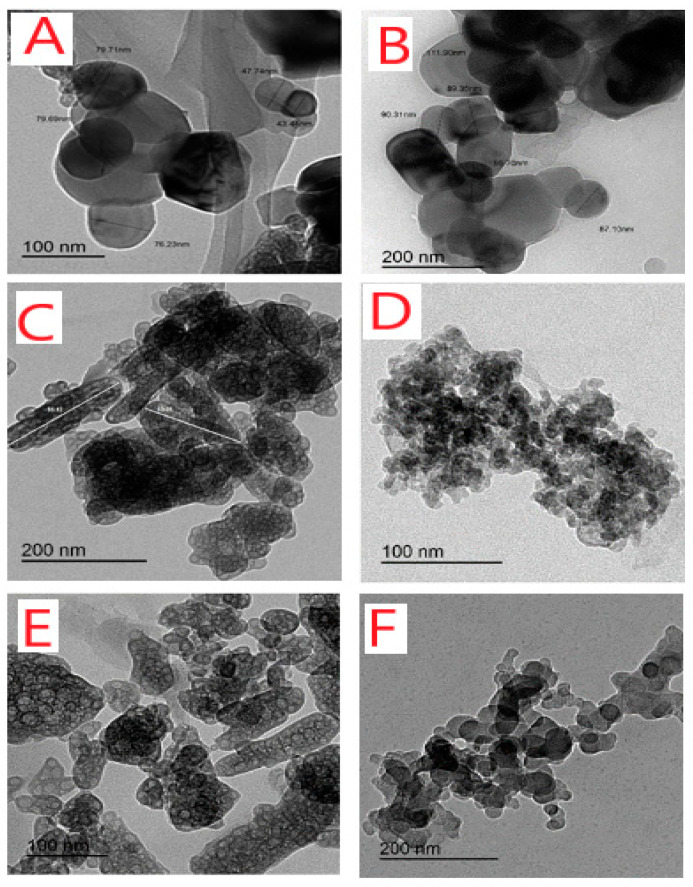
TEM images of (**A**) Mg(II), (**B**) Ca(II), (**C**) Cr(III), (**D**) Zn(II), (**E**) Cu(II), and (**F**) Se(IV) STG complexes.

XRD, SEM, and TEM analyses showed the crystalline nature of the metal complexes. The samples of the solid STG complexes were characterized at room temperature by X-ray diffraction by using Cu Kα radiation. A crystalline form of STG showed an X-ray powder diffraction pattern with peaks at the 2-theta of 13.7, 18.0, 22.6, 25.7, and 27.0 degrees. The diffractograms of the Mg^2+^, Ca^2+^, Cr^3+^, Zn^2+^, Cu^2+^, and Se^4+^ STG complexes showed distinguished patterns at (14.94, 22.75, 25.35, 26.59, 30.27, 32.62, 40.22, 49.40, and 53.10 degrees), (22.75, 25.35, 26.61, 28.59, 30.28, 32.55, 32.96, 36.11, 40.15, 41.08, 47.42, 49.27, and 53.13), (22.43, 23.11, 25.45, 26.66, 28.67, 30.30, 32.87, 36.22, 40.17, and 53.09 degrees), (15.21, 22.75, 26.58, 28.51, 30.21, 32.75, 40.22, 49.27, and 52.96 degrees), (16.65, 21.11, 22.06, 22.54, 25.22, 26.46, 28.64, 28.64, 30.11, and 32.68 degrees), and (16.24, 19.39, 20.42, 22.54, 25.56, 26.63, 30.27, 32.68, 36.18, and 53.10 degrees), respectively. Sharp, intense, and strong Bragg diffraction lines in the diffractograms of STG complexes were well-characterized and presented well-organized structures. The crystalline size of synthesized STG complexes was calculated using the Scherrer formula: D = kλ/βCosθ [32], where k is a constant and equal to 0.94, λ is the used wavelength of X-ray (0.154 nm), and β is a full width at half maximum peak of the XRD pattern [33]. The particle size of STG complexes was found to be within 50–120 nm. It was observed that crystalline size is different for these complexes due to changes in metal ions. SEM images of the Mg(II), Zn(II), Cu(II), and Se(IV) STG complexes are shown in Figure 3A–D. From this figure, it can be see that the average lengths of the grains for these complexes are in the range of 5–50 μm. The surface morphology was found to change with changes in metal ions, with the images having large numbers of irregularly shaped grains and smaller numbers of regularly shaped grains. It is quite clear from the results that the average grain size estimated by SEM was larger than the average grain size measured by XRD. TEM images of the Mg(II), Ca(II), Cr(III), Zn(II), Cu(II), and Se(IV) complex nanoparticles resulting from the complexes between metal chloride salts and two STG molecules are display in Figure 4A–F. After chelation, the particle size was found to be within the range of 50–100 nm with spherical black spots, which was in good agreement with X-ray powder diffraction data.

#### 3.1.6. Thermogravimetric Study

**Figure 5:**
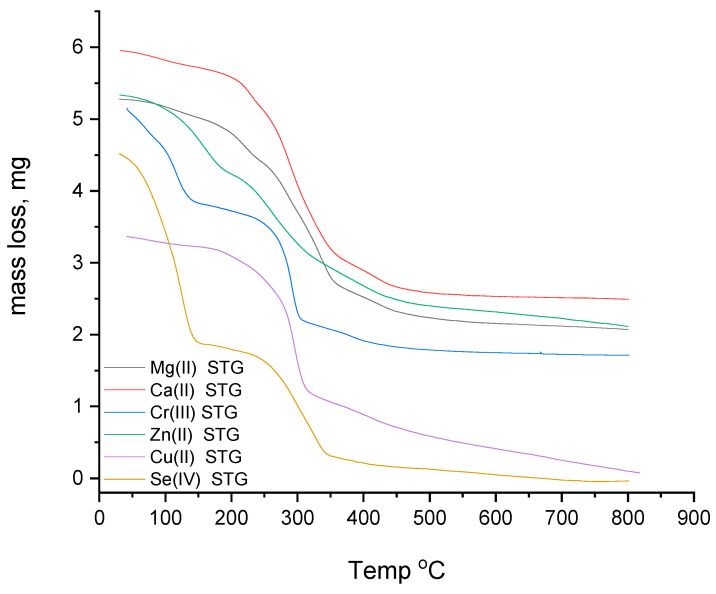
TGA curves of Mg(II), Ca(II), Cr(III), Zn(II), Cu(II), and Se(IV) STG complexes.

The degradation profiles and thermal stabilities of the Mg^2+^, Ca^2+^, Cr^3+^, Zn^2+^, Cu^2+^, and Se^4+^ STG complexes were investigated by TGA measurements. Figure 5 and Table 3 show TGA thermograms and thermal degradation patterns of the complexes, respectively. Cr(III)-STG showed a better thermal stability (686 °C) than the other STG complexes. The TGA thermograms indicate that the Mg(II), Ca(II), Zn(II), Cu(II), and Se(IV) STG complexes are thermally stable up to 662, 674, 565, 654, and 330 °C, respectively. It was observed that the calcium(II) STG complex; the Mg(II), Cr(III), and Cu(II) STG complexes; and the Zn(II) and Se(IV) STG complexes are thermally decomposed within four, three, and two endothermic steps, respectively. The thermogram of the STG complexes indicates that the most stable final product of the thermal degradation was metal oxide polluted with carbon atoms, except for that of selenium(IV), where there were no residual products because of the sublimation behavior of selenium at high temperatures.

### 3.2. Biology Part

#### 3.2.1. Results

The untreated diabetic group (STZ group) had the highest blood glucose and Hb A1c levels of all groups. In addition, this group had the lowest insulin and c-peptide levels of all groups as shown in Table 4.

Compared to the control group, group 3 (STZ and STG) and group 4 (STZ and STG/Cu) had significantly higher blood glucose levels, and they had significantly lower insulin and c-peptide levels. However, there were no statistically significant differences between the control group and group 4 (STZ and STG/Cu) in Hb A1c. There were no statistically significant differences between the control group and groups 5, 6, 7, 8, and 9 regarding blood glucose, Hb A1c, insulin, and c-peptide levels. 

##### Oxidative Stress Enzymatic and Non-Enzymatic Biomarkers

The untreated diabetic group (STZ group) had the highest MDA level of all groups. In addition, it had the lowest GPX, CAT, and SOD levels of all groups as shown in Table 5. Compared to the control group, group 3 (STZ and STG) and group 4 (STZ and STG/Cu) had significantly higher MDA levels, and they had significantly lower GPX and CAT levels. However, there were no statistically significant differences between the control group and group 4 (STZ and STG/Cu) in SOD. There were no statistically significant differences between the control group and groups 5, 6, 7, 8, and 9 regarding GPX, CAT, MDA, and SOD levels.

##### Histological Examination

**Figure 6:**
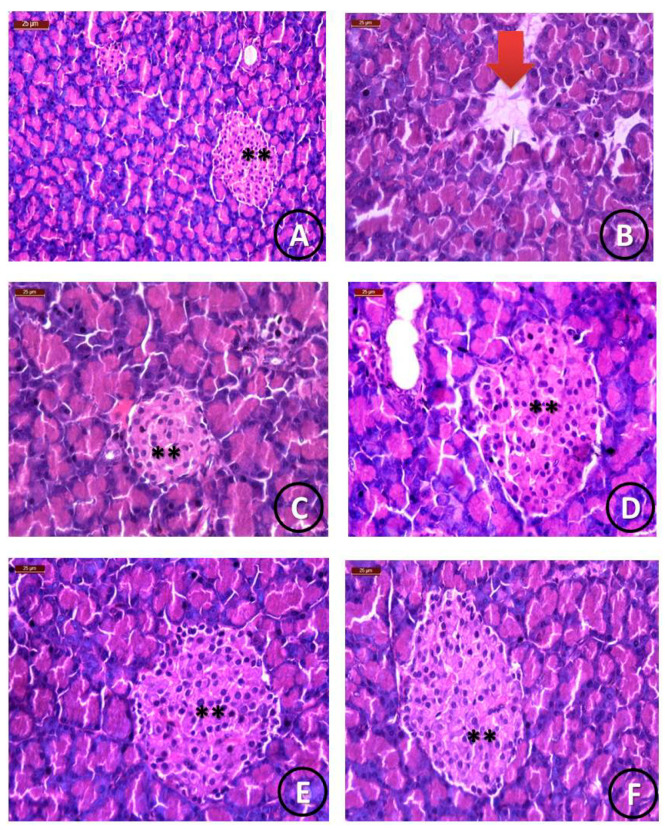

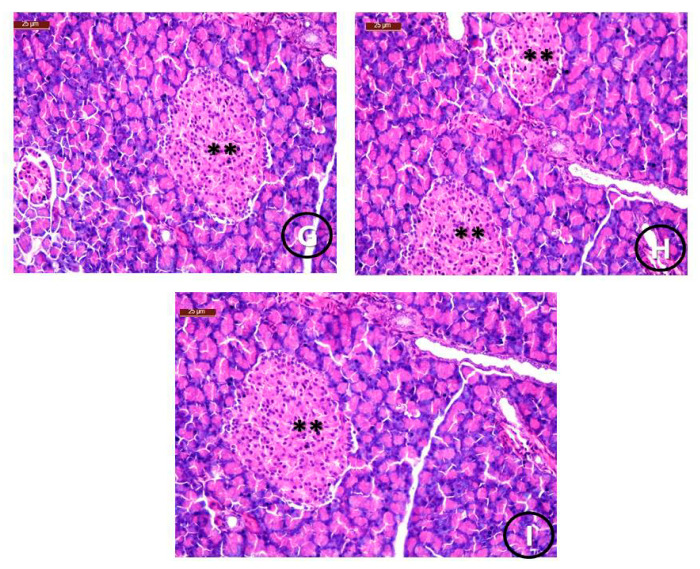
Histological photomicrographs of pancreatic tissues. (**A**) Normal pancreatic parenchyma and the normal appearance of islets of Langerhans (**) (scale bar = 25 µm). (**B**) STZ group showing detached pancreatic parenchyma (**) with the disappearance of islets of Langerhans (red arrow) (scale bar = 25 µm). (**C**) STZ and STG group showing the restoration of detached pancreatic parenchyma with moderately sized islets of Langerhans (**) (scale bar = 25 µm). (**D**–**I**) STZ and Mg, Cu, Zn, Ca, Cr, and Se/STG groups showing normal pancreatic parenchyma (with the appearance of islets of Langerhans (**) that were greatly enlarged in groups D and F) and enlarged pancreatic islets in group H, which was treated with the Cr/STG novel complex (scale bar = 25 µm).

Figure 6 shows a photomicrograph of the control group, which reveals that pancreatic tissues showed the normal appearance of islets of Langerhans. Figure 6B shows an STZ-treated group with detached pancreatic parenchyma and the disappearance of islets of Langerhans (red arrow). Figure 6C shows the STZ and STG group with the restoration of detached pancreatic parenchyma with moderately sized islets of Langerhans. Figure 6D–F shows the STZ and Cu/STG, Mg, and Zn group with normal pancreatic parenchyma and the appearance of highly enlarged islets of Langerhans. The histological index scoring, used to clarify the pancreatic structural changes, is shown in Table 6.

#### 3.2.2. Discussion

Interestingly, a key challenge in the use of STG for the long-term control and management of type II DM is its adverse effects on the exocrine part of pancreatic tissues, including the induction of acute pancreatitis [34]. Due to the clinical significance and seriousness of pancreatitis, the present study was designed to assess the acute biological effects of STG treatment on exocrine and endocrine pancreatic tissues, as well as to investigate the synergistic and therapeutic action of novel STG–metal complexes.

This study concerned the use of STG and its metal complexes Cu/STG, Mg/STG, and Zn/STG in the treatment of diabetic rats induced by STZ. We tried to reveal the pros and cons of each treatment separately and in combination, as well as the possible improving effect of metals after their addition to STG. A novelty in the current study was the use of novel STG complexes in treatments against diabetic pancreatic complications.

The untreated diabetic animals suffered from increases of blood glucose and Hb A1c levels, as well as decreases of insulin and fasting C-peptide levels. Additionally, pancreatic MDA levels were increased, and all the measured antioxidant enzymes (CAT, SOD, GRx, and GST) significantly decreased. Hyperglycemia leads to severe oxidative stress damage that further accelerates the development of diabetes mellitus. The histological examination of pancreatic tissues revealed reductions in the number and size of the Langerhans islets, as well as their disappearance in major fields of examination with the detachment of pancreatic parenchyma.

In the current study, treatment with metal complexes (Cu, Mg, and Zn) of STG revealed significant improvements in all above-mentioned parameters compared to the untreated diabetic group. 

This study produced results that corroborate many of the findings of the previous work of Kelany et al. [35], who reported that an STG–insulin combination better produced hypoglycemic and protective effects and ameliorated oxidative stress than either drug alone. This combination might have clinical efficacy in uncontrolled type 2 diabetes and confirms the obtained results, which showed that the used metals ameliorate the anti-diabetic effects of STG and may enhance its antioxidant activities.

STZ destroys some of the β-cells of islets of Langerhans, thus resulting in increases of blood glucose levels and thus insulin secretion inhibition. These destructive effects are mostly due to the reduction of glucose entry into adipose tissues and muscles, as well as the increase of glycogen breakdown and glucose production caused by the hepatic tissues [36]. STZ also exhibited reductions of pancreatic islets through the excessive production of reactive oxygen species. These current findings coincide with previously published results [37].

In the current study, STG–metal complexes were able to increase the activity of antioxidant enzymes and enhance antioxidant capacity. This antioxidant effect could be attributed to decreasing lipid peroxidation [38]. This finding was in accordance with that of Bao et al. [39], who attributed the antioxidant effects of STG due to DPP-4 inhibitors that increase GLP-1 levels, since this effect could be blocked by a GLP-1 receptor antagonist. Additionally, GLP-1 was found to activate an AMP-activated protein kinase that, in turn, enhanced the activity of the antioxidant enzymes and lead to a significant improvement of oxidative stress—an effect that was duplicated due to the interaction of the chelated metals and STG.

In humans, most obese individuals with insulin-resistance have shown increases of their insulin secretion and are non-diabetic [40]. Recently, β-cell dysfunction was found to participate in the development and progress of diabetes mellitus due to the failure of the β-cells to compensate for the release of insulin during the insulin resistance process [41]. Impaired β-cell function and insulin resistance are considered to be the hallmarks of type II DM pathogenesis [42]. An STZ, high-fat diet-fed (STZ/HFD) experimental type 2 DM rat model was used to mimic the diabetes mellitus pathology; it was found that HFD induced insulin resistance and a low dose of STZ caused the partial impairment of β-cell function [43].

Our results regarding the effects of STG on pancreatic tissues and the novel effect of STG–metal complexes in controlling STZ/HFD-induced diabetes mellitus in rats showed that there were great differences in the biochemical and histological findings obtained from the diabetic rats orally receiving STG and those receiving STG–metal complexes (Cu, Mg, or Zn), and the best improvement was recorded in the Zn/STG treated diabetic group.

We expect that the great improvement in the diabetic group treated with Zn/STG can be attributed to the importance of zinc as a major element for growth that plays a major role in cellular division. In mammalian pancreatic tissues, Zn is an essential element for the correct secretion and action of insulin in β-cells [44].

Insulin is stored inside secretory vesicles, where two zinc ions coordinate six insulin monomers to form a hexameric structure upon which maturated insulin crystals are based. Changes in zinc levels in pancreatic tissues have been found to be associated with diabetes mellitus. As such, the relationship between zinc and insulin is undoubtedly critical and essential to normal β-cell function. When the exocytosis of insulin occurs, insulin granules fuse with β-cells and release their contents, i.e., insulin and an amount of free zinc, into the extracellular space and local circulation [44]. This evidence indicates the great success of our novel Zn/STG complex in alleviating a diabetic state and improving β-cells size and effectiveness, as noticed in the histological sections of pancreatic tissues.

We also found that our novel Mg/STG, Cu/STG, and Zn/STG complexes can induce decreases of hyperglycemia and improvements of the antioxidant activities of pancreatic tissues homogenates, and these ameliorative effects may be attributed to the SOD enzyme—either the manganese-containing MnSOD (present in the mitochondria) or the dinuclear Cu/Zn-SOD (present in the cytosol and extracellular space)—that performs the role of superoxide detoxification in normal cells and tissues. SOD deficiency has been shown to be associated with development of inflammatory processes [45], and our novel STG–metal complexes greatly improve SOD enzymes levels, thus providing great potency for the antioxidant cellular defense systems of pancreatic tissues and improving the secretion and effect of insulin.

Attempts to use the SOD enzyme itself as a therapeutic agent have been partially successful in animals, but not in humans [46]. Pharmacokinetic problems, including delivery problems and the short half-life of SOD in the blood, are major obstacles to the use of antioxidant enzymes in humans. Thus, we tried to use an anti-diabetic drug (STG) in combination with small-molecule metals (Cu, Mg, and Zn) to increase the hypoglycemic effect of STG and mimic that of natural enzymes such as SOD, which may have similar chemistry and thus be useful in treating diseases under safe biological conditions, low toxicity, and favorable bio- distribution.

The present findings further support the previously reported idea that several mechanisms may be involved in the pathogenesis of diabetes mellitus, such as the accumulation of AGEs and increases of the oxidative stress pathway, as confirmed in the current study in the untreated diabetic group [47]. These oxidative stress pathways cause an imbalance in the mitochondrial redox state of a cell and lead to the excessive formation of reactive oxygen species. Moreover, hyperglycemia causes multi-degeneration through increased oxidative stress, which alters the metabolism and dysfunction in diabetic conditions [48].

Oxidative stress is not only a secondary manifestation of diabetes mellitus but also the first cause of a series of complications involved in diabetes mellitus [38]. The present findings agree with this characterization and are supported by our use of novel STG–metal complexes that may present a proper therapeutic approach to prevent the complications of diabetes, wherein we concentrate on early glycemic control and reducing oxidative stress.

Manganese (Mn) is a major nutrient that is very important to metabolism, especially that of proteins, and cellular protection from oxidative stress and free radicals. Mn is also a significant biological metal due to its presence in the active center of many antioxidant enzymes. Recently, many bioinorganic chemists have developed novel metallodrugs with manganese-based compounds because previous studies had demonstrated that Mn^2+^ supplementation can lower markers of oxidative stress and endothelial dysfunction, thereby lowering diabetes mellitus pathophysiological complications, as confirmed in the current study regarding the complexation of STG with Mn [49].

Oxidative stress is not only a manifestation of diabetes but also the first cause of the cascade of complications involved in diabetic pathology. The present findings agree with this idea and are supported by the current conviction that the proper therapeutic approach with novel STG–metal complexes to prevent complications of diabetes is to concentrate on glycemic control and decreasing oxidative stress [50]. Some of these pathophysiologic mechanisms are potentially modifiable by DPP-IV inhibition [50,51,52]. In addition, Salcedo et al. [53] reported that GLP-1-based anti-diabetic drugs can be an effective new strategy to not only regulate blood glucose but also decrease the apoptotic cellular death of pancreatic β-cells, which is in complete agreement with the current findings.

The main goals of anti-diabetic drugs are to decrease blood glucose levels and to minimize the pathophysiological complications of diabetes mellitus [54]. However, the response to these drugs can show differences due to the multifactorial nature and aspects of type II diabetes mellitus pathophysiology [52].

Calcium intake is inversely associated with the development of type II diabetes, and the benefit of this nutrient appear to be additive [53]. For calcium, intake from supplements rather than from diets is significantly associated with a lower risk of type 2 diabetes. To our knowledge, this is the first prospective study to report complexations between STG and metals (Ca, Mg, Zn, Cr, Cu, and Se), as well as their effects on calcium intake and risk of incident type 2 diabetes, and it is the first to examine the combined effects of this novel Ca/STG complex on diabetes risk. 

Chromium is an essential mineral that is thought to be necessary for normal glucose and lipid homeostasis [55]. Chromium (III) is known as a glucose tolerance factor when in a complex, and it is considered to be biologically active. Severe chromium deficiency is known to cause reversible insulin resistance and diabetes [56]. Manufacturers have aggressively promoted the benefits of chromium in the prevention and treatment of insulin resistance and its associated conditions (type II diabetes, dyslipidemia, and cardiovascular disease), a strategy that agrees with the obtained results that confirmed the role of Cr/STG in reducing blood glucose levels and improving antioxidant enzyme capacities, as well as examining the histological structures of the pancreatic tissues.

Other factors that confirm the role of Se in alleviating the pathogenesis of diabetes mellitus is the participation of minerals in the pathogenesis of insulin resistance and their involvement in the synthesis of insulin [57]. Se is an anti-inflammatory and antioxidant micronutrient [56] that is essential for the insulin-signaling pathway, and it has great role in insulin-resistance mechanisms. Evidence has shown that suitable Se concentrations play key roles in the secretion of insulin and its actions.

The finding broadly supports the work [57], the authors of which suggested a quercetin/sitagliptin combination as a promising therapeutic for the attenuation of doxorubicin-induced testicular toxicity in rats, as well as that the main mechanism involved in such an effect may be due to the combined antioxidant and anti-inflammatory properties of both agents, thus proving the idea that complexation between STG and other metals can enhance their capacities.

The oral administration of STG and STG–metal complexes (either Mg, Cu, Zn, Ca, Cr, or Se) at the tested doses significantly decreased blood glucose levels and led to great improvements in glycemic control, additionally evidenced by the amelioration of the levels of insulin. However, the STG–metal complexes led to the better control of blood glucose levels than STG alone. This could explain the potential synergistic effect of STG on blood glucose homeostasis when combined with metals [58].

## 4. Conclusions

Our results confirmed that all the used STG–metal complexes were efficient and safe for the treatment of hyperglycemia and oxidative injury induced by diabetes mellitus, with more antioxidant potency and insulin promotion in the case of treatment with both Zn and Se/STG complexes and greater improvements of glycemic state with the Cr/STG complex. These results open new avenues to develop therapeutic strategies for diabetes mellitus and, potentially, the prevention of its associated complications.

## Figures and Tables

**Table 1:** Analytical data of the STG complexes.

Complexes	Molecular Weight	Elemental Analysis % Found (Calcd.)			
		C	H	N	M
[Mg(STG)_2_(Cl)_2_]·6H_2_O	1017.94	37.46 (37.76)	4.11 (4.16)	13.29 (13.76)	2.30 (2.39)
[Ca(STG)_2_(Cl)_2_]	925.62	41.50 (41.52)	3.20 (3.27)	15.10 (15.13)	4.21 (4.33)
[Cr(STG)_2_(Cl)_2_]Cl·6H_2_O	1081.08	35.48 (35.55)	3.90 (3.92)	12.93 (12.96)	4.80 (4.81)
[Zn(STG)_2_(Cl)_2_]	950.92	40.39 (40.42)	3.08 (3.18)	14.66 (14.73)	6.83 (6.88)
[Cu(STG)_2_(Cl)_2_]_2_·H_2_O	985.12	39.00 (39.02)	3.44 (3.48)	14.20 (14.22)	6.42 (6.45)
[Se(STG)_2_(Cl)_2_]Cl_2_	1035.41	37.09 (37.12)	2.90 (2.92)	13.50 (13.53)	7.61 (7.63)

**Table 2:** Infrared spectral bands and assignments of STG and its complexes.

Assignments	Compounds						
	STG	Mg(II)	Ca(II)	Cr(III)	Zn(II)	Cu(II)	Se(IV)
N–H stretching	3357	3300	3316	3340	3341	3305	3340
C–H stretching	3059 2917 2850	2918 2850	2917 2849	2919	2918 2850	2923 2854	2918 2851
C=O stretching	1669	-	-	-	-	-	-
C=N stretching	1634	1645	1634	1652	1635	1644	1646
NH2 bending	1609	-	-	-	-	-	-
C–C and C–N stretching	1556 1514 1426	1516	1518 1426	1515 1427	1579 1514 1424	1518 1426	1517 1426
CH in plane bending	1324 1274	1335 1311 1275	1336 1277	1315	1335 1274	1372 1335 1275	1315
C–F stretching	1146 978	1138 1015	1056	1159 1013	1107 1013	1140 1048	1107 1013
CH out-of-plane bending	913 881 844 725	889 848 665	891	950 897 664	894 667	845 621	951 897 664
M–O stretching	-	504	515	556	557	558 515	555 519
M–N stretching	-	446	476	445	477	466 445	440

**Table 3:** Thermal data of the STG complexes.

Complexes	Steps	Temp. Range (°C)	DTA Peak (°C)	TGA Weight Loss (%)		Assignments
				Calc.	Found	
Mg(II) STG	1st	30–250 250–800	84, 222 662	17.58	17.50	6H_2_O and Cl_2_
	2nd			50.50	50.28	2STG molecule
	Residual			31.92	32.22	MgO and polluted carbon
Ca(II) STG	1st	30–250 250–800	80, 225 425, 674	7.67	7.45	Cl_2_
	2nd			56.33	56.47	2STG molecule
	Residual			36.00	36.08	CaO and polluted carbon
Cr(III) STG	1st	30–300 300–800	74, 294 686	19.84	19.60	6H_2_O and ³/²Cl_2_
	2nd			47.10	47.14	2STG molecule
	Residual			33.06	33.26	½Cr_2_O_3_ and polluted carbon
Zn(II) STG	1st	30–120 120–800	78 565	7.47	7.35	Cl_2_
	2nd			58.20	58.44	2STG molecule
	Residual			34.06	34.21	ZnO and polluted carbon
Cu(II) STG	1st	30–350 350–800	184, 294 654	10.86	10.60	2H_2_O and Cl_2_
	2nd			55.11	55.30	2STG molecule
	Residual			34.03	34.10	CuO and polluted carbon
Se(IV) STG	1st	30–800	90, 330	100	100	2Cl_2_ and 2STG
	Residual			0.0	0.0	Selenium metal sublimated

**Table 4:** Comparison of the studied groups in blood glucose, insulin, HbA1C, and fasting serum C-peptide levels.

Groups	Blood Glucose (mg/dL)	Insulin (uIU/mL)	HbA1C (mmol/mol)	Fasting Serum C-Peptide (ng/mL)
Group 1 (Control)	87.4 ± 1.9	25.1 ± 0.6	3.2 ± 0.3	4.2 ± 0.1
Group 2 (STZ)	377.3 ± 4.0 *	4.2 ± 0.3 *	9.2 ± 0.2 *	0.5 ± 0.06 *
Group 3 (STZ and STG)	141.6 ± 4.0 *#	18.9 ± 1.3 *#	5.0 ± 0.5 *#	2.6 ± 0.3 *#
Group 4 (STZ and STG/Cu)	130.8 ± 3.8 *#	19.8 ± 1.5 *#	3.0 ± 0.2#	3.3 ± 0.3 *#
Group 5 (STZ and STG/Mg)	128.98 ± 2.58 #	20.58 ± 1.08 #	3.1 ± 0.08 #	3.80 ± 0.6 #
Group 6 (STZ and STG/Zn)	95.4 ± 2.9 #	23.5 ± 0.6 #	3.3 ± 0.5 #	4.1 ± 0.4 #
Group 7 (STZ and STG/Ca)	110 ± 3.58 #	22.39 ± 2.01 #	4.05 ± 0.36 #	2.50 ± 0.63 #
Group 8 (STZ + STG/Cr)	90.24 ± 3.68 #	23.68 ± 1.88 #	3.01 ± 0.69 #	3.98 ± 0.87 #
Group 9 (STZ and STG/Se)	100.69 ± 2.69 #	21.02 ± 1.69 #	3.68 ± 0.39 #	3.21 ± 0.63 #
*p*-value	<0.001 (HS)	<0.001 (HS)	<0.001 (HS)	<0.001 (HS)

Data are presented as mean ± SD. * Significant difference compared to control group; # Significant difference compared to STZ group. HS: highly significant.

**Table 5:** Comparison of the studied groups in pancreatic GPX, CAT, MDA, and SOD levels.

Groups	GPX (U/g)	CAT (U/g)	MDA (U/g)	SOD (U/g)
Group 1 (Control)	34.1 ± 3.0	1.7 ± 0.2	3.1 ± 0.2	21.5 ± 1.2
Group 2 (STZ)	7.4 ± 0.2 *	0.24 ± 0.05 *	80.3 ± 1.2 *	5.4 ± 0.3 *
Group 3 (STZ and STG)	20.9 ± 1.5 *#	1.0 ± 0.2 *#	20.0 ± 0.8 *#	14.0 ± 0.8 *#
Group 4 (STZ and STG/Cu)	22.06 ± 0.68 *#	1.03 ± 0.01 *#	17.68 ± 0.85 *#	17.29 ± 1.25 #
Group 5 (STZ and STG/Mg)	24.88 ± 0.45 #	1.45 ± 0.1 #	10.02 ± 1.36 #	18.05 ± 1.59 #
Group 6 (STZ and STG/Zn)	31.0 ± 1.6 #	1.60 ± 0.3 #	7.9 ± 0.7 #	19.9 ± 1.3 #
Group 7 (STZ and STG/Ca)	29.58 ± 1.78 #	1.54 ± 0.02 #	8.05 ± 1.58 #	19.05 ± 1.36 #
Group 8 (STZ and STG/Cr)	32.17 ± 2.36 #	1.68 ± 0.05 #	4.98 ± 1.02 #	20.54 ± 1.98 #
Group 9 (STZ and STG/Se)	30.25 ± 2.58 #	1.34 ± 0.04 #	5.25 ± 1.32 #	19.85 ± 2.05 #
*p*-value	<0.001 (HS)	<0.001 (HS)	<0.001 (HS)	<0.001 (HS)

Data are presented as mean ± SD. * Significant difference compared to the control group; # Significant difference compared to the STZ group. HS: highly significant.

**Table 6:** Histopathological findings in the pancreatic tissues of normal rats, untreated diabetic rats, and diabetic rats treated with either STG, Cu/STG, Mg/STG, or Zn/STG.

Groups	Pancreatic Parenchyma		Islets of Langerhans Size			
	Normal	Detached	Normal	Reduced	Mild	Enlarged
Group 1 (Control)	90%	0%	90%	0%	0%	0%
Group 2 (STZ)	0%	90%	0%	90%	25%	0%
Group 3 (STZ +STG)	80%	25%	80%	0%	80%	80%
Group 4 (STZ+ STG/Cu)	90%	0%	90%	0%	25%	90%
Group 5 (STZ+ STG/Mg)	90%	0%	90%	0%	25%	90%
Group 6 (STZ+ STG/Zn)	90%	0%	90%	0%	25%	90%
Group 7 (STZ+ STG/Ca)	90%	0%	90%	0%	25%	90%
Group 8 (STZ+ STG/Cr)	90%	0%	90%	0%	25%	90%
Group 9 (STZ+ STG/Se)	90%	0%	90%	0%	25%	90%

Data are expressed as percentages of change.

## Data Availability

The data that support the findings of this study are available from the corresponding author upon reasonable request.
